# Supplementary material for: Development of the Nursing Nutritional Care Behaviors Scale (B-NNC) in Italian and Psychometric Validation of Its German Translation in Austria
Source: Nurs Rep. 2025 Apr 28;15(5):146. doi: 10.3390/nursrep15050146 (PMC12113720; doi:10.3390/nursrep15050146)

Table S1. Summary of the included studies of the scoping review performed in the conceptualization phase of the scale

Note: Main results are presented by highlighting challenges, components, and practices of nurses and nurse assistants according to the main research question: "What are the challenges, components, and practices related to nutritional care behaviors for older adults performed by registered nurses and nurse assistants, as reported in the published literature?"

Author(s)	Year	Study Design	Location	Main results
**Sevillano-Jiménez, Alfonso et al.**	2022	RCT	Spain	**Challenges**: The study identified a lack of emphasis on nutrition in routine psychiatric care and the need for specialized nursing interventions to address poor dietary habits and high consumption of ultra-processed foods in vulnerable populations.<br><br>**Components:** Effective nutritional care behaviors included individualized dietary education emphasizing prebiotic and probiotic-rich foods (e.g., dairy, fermented foods, fruits, vegetables) and routine monitoring of dietary adherence and anthropometric changes.<br><br>**Practices**: Psychiatric nurses played a pivotal role in delivering tailored dietary advice and ensuring adherence to nutritional plans, showcasing the potential of advanced nursing practices to address dietary

				challenges in older adults with complex health needs.
**Tseng, M.-Y. et al.**	2022	RCT	Taiwan	**Challenges**: Malnutrition significantly impacted the effectiveness of a family-centered care intervention for older adults with cognitive impairment recovering from hip-fracture surgery. Most participants (91%) were poorly nourished post-discharge, which hindered functional recovery outcomes.<br><br>**Components**: The intervention included geriatric nurse-led family training focusing on dietary requirements, home-based rehabilitation exercises, and managing cognitive and behavioral issues. Nutritional assessments were conducted using the Mini-Nutritional Assessment (MNA), and tailored dietary consultations were provided.<br><br>**Practices**: The family-centered care intervention improved physical health, IADLs, and hip muscle strength only for well-nourished participants. This highlighted the critical role of addressing malnutrition as part of

				post-operative care for older adults.
**Ten Cate, Debbie et al.**	2022	Delphi	Netherlands	**Challenges**:<br>The study identified eight key factors influencing nurses' behaviors in providing nutritional care for older adults, including insufficient knowledge, low prioritization, moderate awareness of risk factors, and ambiguous motivation to use guidelines, tools, or pursue training.<br><br>**Components**:<br>Relevant and modifiable factors were:<br>Neutral attitudes toward nutritional care.<br>Limited focus on involving informal caregivers.<br>Strong reliance on medical nutrition rather than comprehensive approaches.<br><br>**Practices**:<br>The expert consensus emphasized the need for targeted strategies to improve nutritional care behaviors, including better training, guideline implementation, and increasing awareness about malnutrition risk factors among nurses in hospital and home care settings.

**Terp, Rikke et al.**	2022	Mixed method	Denmark	**Challenges**:<br>The study addressed insufficient protein-energy intake among older hospital patients, which is a critical concern linked to functional decline, prolonged hospital stays, and higher readmission rates. Barriers included limited patient involvement in nutritional care, inadequate nursing practices for malnutrition management, and technical challenges with using eHealth tools like Food'n'Go.<br><br>**Components**:<br>The Educative Nutritional Intervention (ENI) was developed using the Intervention Mapping framework to: Support patient participation in nutritional care through eHealth (Food'n'Go). Equip nurses with training and education to promote patient engagement in monitoring and meeting dietary requirements.<br><br>**Practices:**<br>The ENI included tailored strategies such

				as bedside teaching for nursing staff, patient categorization based on their competence with Food'n'Go, and materials like user guides and posters. The program emphasized theory-driven behavior change and skills training to improve self-efficacy and address barriers to nutritional care.
**Shirley, Louisa et al.**	2022	Discussion paper	UK	**Challenges:**<br>Eating disorders in older people are often overlooked because symptoms like changes in eating behaviors, weight, and mental health are mistakenly attributed to normal aging or "anorexia of ageing," rather than being recognized as diagnosable conditions.<br><br>**Components**:<br>The study highlights the need for nurses to focus on detection, referral, and support for older adults with suspected eating disorders. It emphasizes that understanding the psychological distress underlying these disorders is critical.<br><br>**Practices**:<br>The authors propose a framework for nurses to assess older adults for potential eating disorders. This includes evaluating changes in

				eating behaviors, mental health, and physical signs, and distinguishing these from normal aging processes.
**Frates, Beth et al.**	2022	Literature review	USA	**Challenges**:<br>Nutrition counseling and behavior change remain underutilized in healthcare, with gaps in addressing the unique needs of different age groups and vulnerable populations. Despite advancements in nutrition science, implementation at individual and population levels still faces significant barriers.<br><br>**Components**:<br>Effective counseling requires clear, multifactorial language to educate and empower patients.<br>Targeted Strategies: Approaches vary by population:<br>Young adults: Use of video games and social media to engage.<br>Older adults: Focus on altering food consistency and ensuring adequate nutrient intake.<br>Vulnerable populations: Special attention to addressing malnutrition risks.

				**Practices**:<br>Healthcare providers, including physicians and nurses, play a key role in lifestyle counseling. The study emphasizes the importance of integrating behavior change strategies into healthcare operations to promote healthy eating patterns.
**Eaton, Maggie et al.**	2022	Qualitative study	USA	**Challenges**:<br>Nutrition counseling is recognized as critical in primary care but is underutilized due to barriers like time constraints, lack of resources, and patient resistance to behavior change.<br>Nurse practitioners (NPs) face challenges in developing self-efficacy and managing the emotional aspects of nutrition counseling.<br><br>**Components**:<br>Five key themes emerged from NPs' experiences:<br>Role in Primary Care: NPs see nutrition counseling as integral to their practice.<br>Developing Self-Efficacy: NPs need confidence and skills for effective counseling.<br>Beyond Information: Counseling involves emotional support and

				personalized approaches.<br>Emotional Dimensions: Food-related behaviors are tied to emotions, requiring empathy in counseling.<br>Barriers to Change: Patient noncompliance and limited resources hinder counseling efforts.<br>**Practices**:<br>NPs understand the importance of nutrition counseling but highlight the need for better tools, training, and strategies to overcome barriers and improve patient outcomes.
**Gartrell, Kyungsook et al.**	2020	Cross-sectional study	Republic of Korea	**Challenges**:<br>Nurses' ability to effectively use eHealth information is critical for improving patient care but is not always fully developed.<br>There is limited evidence on the relationship between eHealth literacy and nursing performance or health-promoting behaviors.<br><br>**Components**:<br>The study identified a three-factor structure of the eHealth Literacy Scale (eHEALS):<br>Awareness of internet health resources.<br>Skills to access these

				resources.<br>Evaluation of the quality of information.<br>These factors were significantly associated with better nursing performance and interpersonal relationships.<br><br>**Practices**:<br>Nurses with higher eHealth literacy demonstrated improved stress management, health responsibility, spiritual growth, and interpersonal relationships.<br>However, eHealth literacy was not significantly associated with physical activity or nutrition, indicating barriers like time constraints or shift work.
**Dellafiore, Federica et al.**	2021	Multi-method	Italy	**Challenges**:<br>Malnutrition among older adults remains a significant issue, often exacerbated by nurses' inadequate attitudes and limited confidence in delivering nutritional care.<br>Barriers include insufficient knowledge, underutilization of assessment tools, and challenges in providing individualized care due to workloads and systemic limitations.

				**Components**:<br>The study developed the Self-Efficacy Scale for Nursing Nutrition Care (SE-NNC) to measure nurses' confidence in providing nutritional care.<br>The scale encompasses 27 items across three domains:<br>Knowledge: Understanding nutritional care principles.<br>Assessment and Evidence Utilization: Conducting assessments and leveraging evidence-based tools.<br>Care Delivery: Providing individualized, patient-centered nutritional interventions.<br><br>**Practices**:<br>The SE-NNC demonstrated strong psychometric properties, including content validity, construct validity, and internal consistency.<br>It provides a framework for identifying gaps in nurses' self-efficacy and tailoring educational interventions to improve their skills and confidence in nutritional care.

**Pentecost, Claire et al.**	2020	Literature review	UK	**Challenges**:<br>The study highlights gaps in the delivery of fundamental nursing care, particularly for nutrition, hygiene, mobility, and elimination, due to inadequate leadership, lack of training, and inconsistent organizational priorities.<br>Patients often experience fragmented care, while nurses report challenges related to time constraints, insufficient staff, and systemic barriers in prioritizing fundamental care tasks.<br><br>**Components**:<br>Key components of effective nursing care include nurse leadership, partnerships with patients, and organizational practices.<br>Nursing care behaviors focus on assisting patients to maintain independence, addressing their individual needs, and fostering collaboration through patient-centered care approaches.

				**Practices**:<br>Effective practices include promoting mealtime assistance and nutritional support and creating opportunities for patients to participate in self-care. Leadership involves training and empowering nurses, facilitating teamwork, and aligning organizational policies to support high-quality care.
**Liu, Wen et al.**	2019	RCT	USA	**Challenges**:<br>Residents with dementia in nursing homes often experience inadequate food and liquid intake, leading to malnutrition, dehydration, and poor health outcomes.<br>Staff frequently provide full mealtime assistance without fully engaging residents in eating, which may reduce intake effectiveness and compromise independence.<br><br>**Components**:<br>Eating Performance Cycles: The study focused on characteristics such as eating technique (resident-completed vs. staff-facilitated), food type (solid vs. liquid), and cycle duration.<br>CUED Coding Scheme:

				A novel video-coding tool was used to analyze eating performance cycles and interactions during mealtime.<br><br>**Practices**: Resident-completed cycles were more likely to result in successful intake compared to staff-facilitated cycles, especially with solid food.<br>Liquid food was associated with higher intake rates than solid food, particularly when cycles lasted longer.<br>Longer durations of staff-facilitated cycles increased the likelihood of intake for residents needing assistance.
**Głąbska, Dominika et al.**	2015	Cross-sectional study	Poland	**Challenges**: Diabetology nurses exhibited poor nutritional behaviors, including low intake of fish, legumes, milk, and dairy products and insufficient moderation in sugar and sweets consumption.<br>Nurses lacked regularity and diversity in meal patterns, even though they were responsible for educating diabetic patients about proper nutrition.<br>No correlation was found between years of working with diabetic

				individuals and adherence to recommended nutritional goals.<br><br>**Components**:<br>The study assessed adherence to nutritional goals using a self-reported scoring system where participants rated their frequency of following dietary recommendations as "always," "sometimes," or "never."<br>Nurses scored significantly lower than the control group in regularity and diversity of meals.<br><br>**Practices**:<br>Despite their role in patient education, nurses demonstrated inadequate adherence to recommended nutritional practices, reflecting a gap between professional knowledge and personal behaviors.
**Kuehlmeyer, Katja et al.**	2015	Cross-sectional study	Germany	**Challenges**:<br>Nonverbal behavior during feeding for residents with advanced dementia is crucial yet inconsistently interpreted by nursing staff.<br>Discrepancies in interpreting behaviors such as refusal to open the mouth or showing

				pleasure during eating can complicate decisions about artificial nutrition and hydration (ANH).<br><br>**Components**:<br>The study surveyed nursing staff in residential care homes to evaluate how nonverbal behaviors influence decisions about ANH. Nonverbal expressions, such as not opening the mouth or slapping away a nurse's hand, were frequently observed during hand feeding.<br><br>**Practices**:<br>Many participants considered residents' behavior as an important factor in ANH decisions. Behaviors like pleasure during eating were interpreted as a will to live, while aversive behaviors were often linked to discomfort. Cultural and personal factors, such as the religious affiliation of nursing homes, influenced interpretations of residents' behavior.
**Pedersen, Preben U. et al.**	2012	Quasi experimental design	Denmark	**Challenges**:<br>Malnutrition affects approximately 40% of hospitalized patients,

				contributing to complications, increased mortality, and reduced quality of life. Nurses often have insufficient knowledge and neutral or negative attitudes toward nutritional care, posing a barrier to implementing nutritional guidelines effectively.<br><br>**Components**:<br>The study evaluated the effect of a systematic training program for Nutritional Nurse Practitioners (NNPs) to implement nutritional guidelines.<br>The training incorporated experimental learning theory and the look, think, act model, which involved gathering baseline data, analyzing findings, and implementing ward-specific interventions.<br>Key topics included assessing nutritional risk, addressing eating difficulties, and tailoring nutritional interventions.<br><br>**Practices:**<br>Training improved nurses' ability to identify and address

				patients' eating difficulties, provide the right food choices, and promote snack intake between meals.<br>Patients in the post-training period reported receiving more appropriate assistance, better food choices, and greater support for eating snacks, which likely increased their nutritional intake.
**Chen, Su-Hui et al.**	2010	Cross-sectional study	Taiwan	**Challenges**:<br>Older Taiwanese adults face poor nutritional status due to age-related changes, inadequate nutritional knowledge, and external health control beliefs.<br>Factors like lower education levels, external control orientations (e.g., belief in luck or fate), and chronic diseases negatively impact nutritional outcomes.<br><br>**Components**:<br>The study integrated Self-Efficacy Theory and Health Locus of Control (HLC) Theory to explore their relationship with nutritional status.<br>Nutritional self-efficacy was measured using the Cardiac Diet Self-Efficacy scale, while nutritional status was

				assessed through the Mini-Nutritional Assessment (MNA) and serum albumin levels.<br><br>**Practices:**<br>Internal HLC (belief in personal control over health) was positively associated with better nutritional outcomes. Chance HLC (belief that health is influenced by luck or fate) negatively affected nutrition self-efficacy and MNA scores, highlighting the need for interventions targeting control beliefs.
**Green, Sue M. & Watson, Roger**	2005	Literature review	UK	**Challenges:**<br>Malnutrition is prevalent in healthcare settings, with up to 40% of hospitalized patients affected.<br>Many nutritional screening and assessment tools lack extensive testing for validity, reliability, sensitivity, specificity, and acceptability.<br><br>**Components:**<br>The study reviewed 35 tools available for use by nurses to assess nutritional risk, focusing on their practicality and evidence of testing. Tools range from simple questionnaires assessing weight and

				dietary intake to more comprehensive instruments incorporating medical and socio-cultural factors.<br><br>**Practices:**<br>Nurses play a key role in conducting nutritional assessments and implementing care plans based on screening results.<br>The study emphasized that tools should be tailored to specific patient populations and contexts to ensure accuracy and utility in clinical practice.
**Cho, Hyunyi & Nadow, Michelle Zbell**	2004	Qualitative study	USA	**Challenges**:<br>Despite funding and resources, schools face significant barriers to creating a healthy nutritional environment due to:<br>Students' preference for unhealthy foods.<br>Insufficient parental and community involvement in fostering healthy eating behaviors.<br>Lack of communication among food service staff, health educators, and teachers.<br>Competing priorities, such as the state's academic assessment system, reduce focus on nutrition programs.

				**Components**:<br>Effective lunch and nutrition education programs require alignment of efforts among superintendents, principals, food service directors, nurses, and health educators. Collaborative strategies are necessary to integrate healthy eating into both school lunches and school-wide nutrition education.<br><br>**Practices**:<br>The study emphasized the need for better communication and coordination among school staff to promote nutritional initiatives. Enhanced leadership interest and support from superintendents and principals were identified as key drivers for successful implementation.
**Perry, L.**	1997	Cross-sectional study	UK	**Challenges**:<br>Significant gaps exist in nurses' knowledge and application of nutritional care, with many relying on guesswork or limited tools such as patient weights, which are often inaccurately recorded.<br>Discrepancies between nurses' stated attitudes, documented activities, and actual practice

				reflect inconsistencies in nutritional assessment and care.<br><br>**Components:**<br>The study explored relationships between nurses' attitudes, knowledge, and behaviors using Ajzen and Fishbein's Theory of Reasoned Action, revealing weak correlations.<br>Factors such as education level, workplace norms, and years of experience influenced nutritional care practices, yet knowledge deficiencies persisted across all groups.<br><br>**Practices**:<br>Most nurses identified nutritional care as part of their responsibilities, but only a minority conducted thorough nutritional assessments or implemented effective interventions. Documentation often lacked actionable nutritional plans or evaluations, highlighting gaps in translating stated responsibilities into practice.

Figure S1. Summary of the included studies by study design

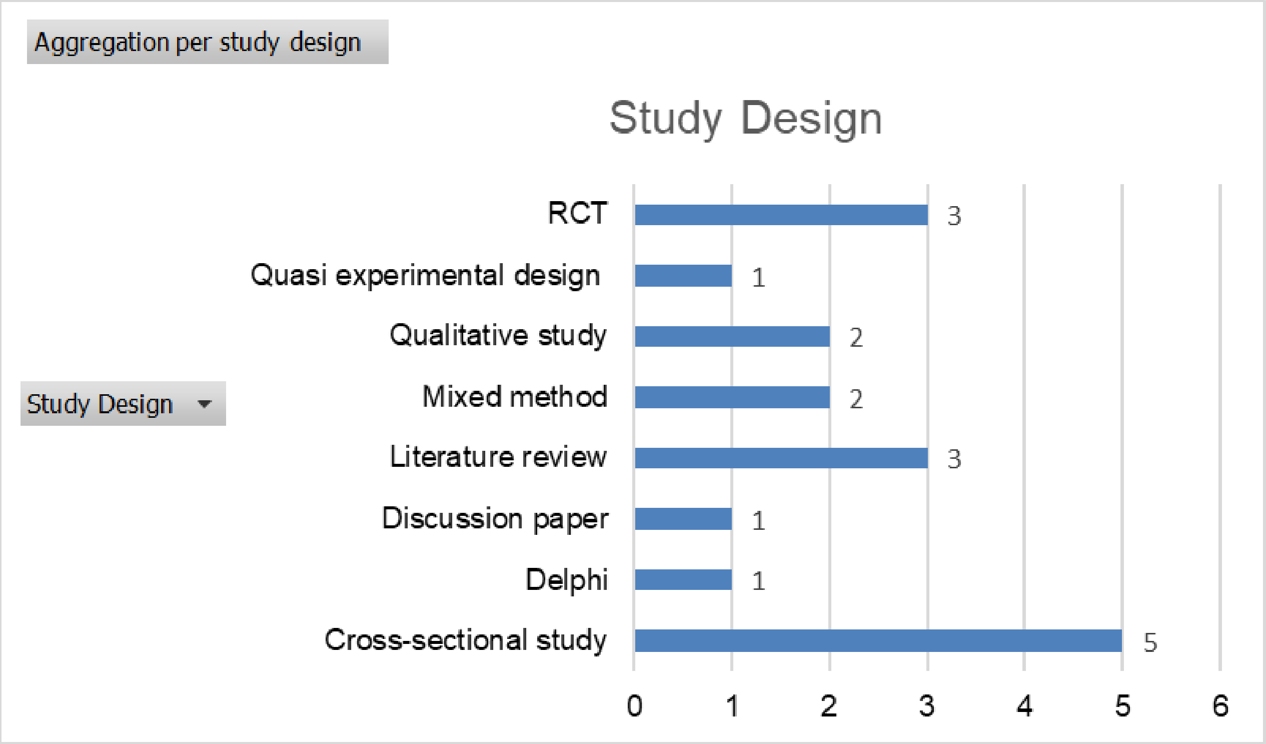

Figure S2. Summary of the included studies by geographic location

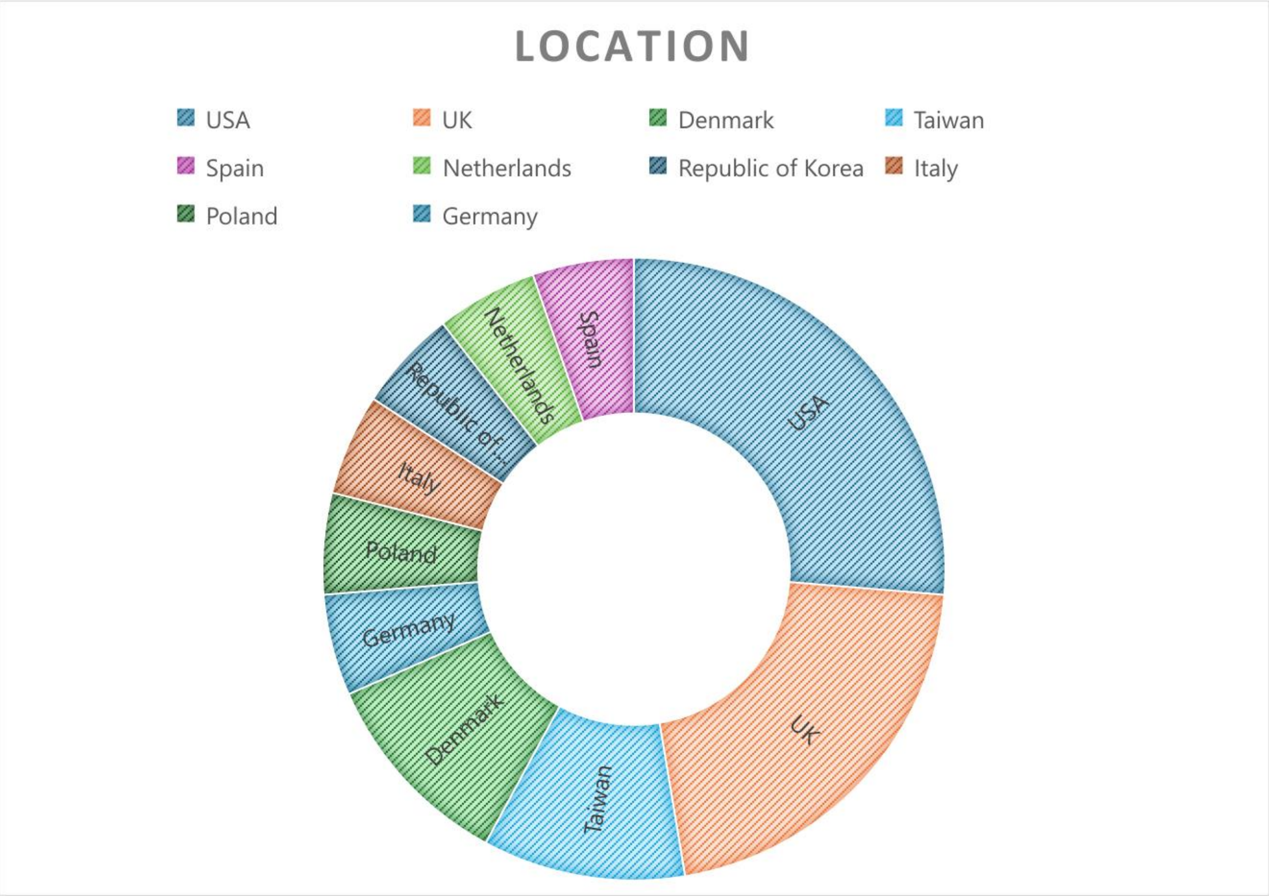

Supplement: Supplementary file 1 [file nursrep-15-00146-s001.zip › nursrep-3553166-supplementary.pdf]
